# Cold wind of change: Associations between organizational change, turnover intention, overcommitment and quality of care in Spanish and Swedish eldercare organizations

**DOI:** 10.1002/nop2.615

**Published:** 2020-09-02

**Authors:** Robert Lundmark, Maria Nordin, Montserrat Yepes‐Baldó, Marina Romeo, Kristina Westerberg

**Affiliations:** ^1^ Department of Psychology Umeå University Umeå Sweden; ^2^ Department of Social Psychology University of Barcelona Barcelona Spain

**Keywords:** care of older people, eldercare, organizational change, overcommitment, quality of care, turnover intentions

## Abstract

**Aim:**

To examine the association between organizational change, turnover intentions, overcommitment and perceptions of quality of care among nurses and nursing assistants employed in eldercare organizations.

**Design:**

A longitudinal survey (baseline, 12‐month follow‐up) was used.

**Methods:**

A panel sample of 226 eldercare employees in Spain and Sweden responded to survey questions concerning organizational change, turnover intentions, overcommitment and perceptions of quality of care. The data were analysed using structural equational modelling.

**Results:**

We found a statistically significant positive relationship between organizational change, employees’ turnover intention and overcommitment. We also found a statistically significant negative relationship between organizational change and perceived quality of care.

## 1 INTRODUCTION

Most countries in the Western world face a shortage of active nursing staff, challenging the general effectiveness, as well as the providence of a high‐quality care (Drennan & Ross, 2019). The shortage of qualified employees, in combination with an ageing population and a high degree of premature turnover, is a challenge for many eldercare organizations (Clausen, Tufte, & Borg, 2014; Stone et al., 2017). Likewise, high levels of burnout among employees and reduced quality of care, in eldercare organizations has been reported (Passalacqua & Harwood, 2012). Therefore, understanding what causes eldercare employees to leave their job, or become absent from work, is vital for the availability and stability of care of older people (Stone et al., 2017; Touarangeau, 2014).

A frequently given reason for eldercare employees’ turnover and absence is dissatisfaction with working conditions caused by factors related to the organization (Clausen et al., 2014; Tourangeau, Patterson, Saari, Thomson, & Cranley, 2017). One organizational factor suggested to be able to generate negative effects on employee health and well‐being is organizational change (de Jong et al., 2016). Organizational change is a broad concept that can involve a wide range of different strategies and actions, including anything from new organizational routines to delocalization, internal reorganization or downsizing (Saksvik et al., 2007).

Organizational change may thus from an employee perspective infer extra stain in the already challenging work environment that constitutes eldercare (van der Borg, Verdonk, Dauwerse, & Abma, 2017). Eldercare organizations are undergoing rapid and significant transformation, with frequent organizational change being the rule rather than the exception; however, studies on employee outcomes of these changes are sparse (von Treuer et al., 2018). The aim of this study is therefore to investigate how organizational change over the last year relates to employee outcomes in terms of turnover intentions, overcommitment (i.e. behaviours associated with burnout) and perceptions of quality of care among nurses and nursing assistants employed in Spanish and Swedish eldercare organizations?

In examining these relationships, the present study contributes by providing knowledge on how eldercare employees cope in a work environment with ongoing organizational change. By understanding what mechanisms leads to turnover, absence (e.g. as a result of overcommitment) and performance (e.g. quality of care), counteractive measures can be taken to ensure a sound working environment, the well‐being of employees and improved care provision. Given the high turnover rate in eldercare (Stone et al., 2017) and general findings of the effects of organizational change on turnover, absence and performance (Jong et al., 2016; Oreg, Vakola, & Armenakis, 2011), this is an important gap to fill.

### 1.1 Organizational change ‐a threat to employee resources

Conservation of Resource Theory (COR; Hobfoll, 1989), has been suggested as theoretical framework that can help explain healthcare employees’ reactions to protect resources that they perceive threatened (Van der Heijden, Mulder, König, & Anselmann, 2017). COR is based on the tenet that individuals strive to obtain, foster and protect valued resources and minimize potential loss of resources. Resources include object resources (e.g. tools for work), personal resources (e.g. skills), condition resources (e.g. experience, autonomy) and energy resources (e.g. knowledge and emotions). COR stipulates that coping strategies are employed to avoid or deal with the stress that occurs when resources are perceived as threatened or lost (Hobfoll, 1989). As employees commonly appraise organizational change as a threat to resources they will, depending on their understanding of the situation, engage in some form of coping (Fugate, Kinicki, & Prussia, 2008).

### 1.2 Organizational change and employees’ turnover intention

As shown in an extensive review on change recipients' reactions, organizational change has previously been positively associated with turnover intentions (Oreg et al., 2011). From a COR perspective, turnover intention can be viewed as a cognitive representation of an escape coping strategy (Fugate et al., 2008). In other words, a way to avoid the perceived negative consequences that organizational changes involves planning to leave could be understood as a way of avoiding being resource depleted. We therefore hypothesis that:

Hypothesis 1 The degree to which eldercare employees in Spain and Sweden perceive that they have been affected by organizational change over the last year will be positively associated with their turnover intentions.

### 1.3 Organizational change and employees’ overcommitment

Overcommitment can also be considered as a coping strategy to handle the threat of loss of resources (Heijden et al., 2017). Originally overcommitment was formulated as part of the effort‐reward imbalance model and seen as a strategy to handle imbalance between effort and reward (Siegrist et al., 2004). However, overcommitment has also been studied as an independent coping strategy outside the effort‐reward imbalance concept (Romeo, Yepes‐Baldó, Piñeiro, Westerberg, & Nordin, 2019). Overcommitment is characterized by excessive job involvement and a desire to control a demanding situation and has been associated with a decrease in well‐being and health among employees (Siegrist et al., 2004).

From a COR perspective, this coping strategy can be seen as based on the assumption that some individuals, when faced with the risk of losing resources, try to compensate by higher levels of effort. In the long run, this will interfere with their time to recover and may lead to resource depletion and the risk of being burned‐out (Heijden et al., 2017). As argued above, when employees appraise organizational change as a threat to resources, they will try to resolve the situation by adopting a coping strategy (Fugate et al., 2008). Alternatively to an escape strategy such as turnover intentions, for some turnover is not an option and if very committed to‐ and engaged in‐, their work, overcommitment may be a more likely strategy. We therefore hypothesis that:

Hypothesis 2 The degree to which eldercare employees in Spain and Sweden perceive that they have been affected by organizational change over the last year will be positively associated with their overcommitment.

### 1.4 Organizational change and employees’ appraisals of quality of care

Appraisals of quality of care have previously been used as both a performance outcome measure to study the influence of different contextual factors in healthcare organizations and as an outcome or nurses’ health (Poghosyan, Clarke, Finlayson, & Aiken, 2010). Quality of care has been related to lower patient satisfaction (Vahey, Aiken, Sloane, Clarke, & Vargas, 2004), as well as 30‐day mortality rates among patients (Tourangeau et al., 2007). However, under the condition of organizational change, we suggest that a coping perspective can shed further light on what affects quality of care.

When eldercare employees appraise organizational changes as a threat to resources, we propose that a coping strategy may be to insert less effort (or not increasing effort as extra effort may be a consequence of organizational change) in providing quality of care. In opposite to overcommitment, this strategy like turnover intention strives towards avoiding being resource depleted. Thus, reducing (or not increasing) efforts to provide good quality of care may be a result of an active coping by employees to save themselves from burnout. In organizational change literature in general, organizational change has been found to have a negative relationship with employee effort, effectiveness and performance outcomes (Oreg et al., 2011). Based on the above we hypothesis that:

Hypothesis 3 The degree to which eldercare employees in Spain and Sweden perceive that they have been affected by organizational change over the last year will be negatively associated with their appraisals of quality of care provided.

## 2 METHOD

### 2.1 Design

A longitudinal survey design was used.

### 2.2 Participants and procedures

Participants for the study were recruited among employees in four Spanish and nine Swedish residential nursing homes for older people. In some of the Swedish organizations, home care services for older people were included in their mission. These organizations were participating in a larger project evaluating conditions in eldercare in these two countries. The organizations were thus not selected specifically based on introducing organizational changes. However, in both countries, the organizations reported varying degrees on changes in terms of relocation; changes of work tasks and/or schedules; internal reorganization; and/or downsizing. In 2016 (T1), paper‐and‐pen questionnaires were sent out to 928 employees, of these, 628 responded (a total response rate of 67.7%). A year later, in 2017, a follow‐up questionnaire was sent out (T2). In total, 226 employees of the respondents at T1 also responded to the questionnaire at T2 (36% of the respondents at T1) and thus constitutes the panel sample of the present study.

Employees in the panel sample had an age of mean = 45.8 (*SD*: 11.4). Most of them were women, 92.5% and 10.2% were first line mangers. Comparing the panel sample with all 628 responding employees at T1, one sample *t* test revealed no statistically significant difference in terms of age (*t* = −0.66, *df* = 599, *p* = .51), managerial position (*χ* ^2^ = 0.85, *df* = 627, *p* = .36) or sex (*χ* ^2^ = 0.02, *df* = 627, *p* = .89), nor in terms of overcommitment (*t* = 0.91, *df* = 622, *p* = .36) or perceived quality of care (*t* = −1.96, *df* = 626, *p* = .06). However, a statistically significant difference was found for turnover intention (*t* = 5.41, *df* = 626, *p* < .01), meaning that all responding employees (*N* = 628) scored higher on turnover intentions compared with the panel sample (*N* = 226) at T1.

### 2.3 Power analysis

A power analysis was completed prior to commencement of the study (Soper, 2020; Westland, 2010), recommending a minimum sample size of 161 respondents.

### 2.4 Measures

Self‐ reported turnover intention, overcommitment and quality of care at T2 were used as dependent variables, controlling for employees’ responses on the same latent variables at T1. Perceived organizational change during the last year, responses gathered at T2, was used as an independent variable. The original scales were translated and back‐translated to Spanish and Swedish following the guidelines of the International Test Commission (2005).

#### 2.4.1 Turnover intention

Turnover intention was measured using the need commitment subscale, comprised of three questions, in the Identification‐Commitment Inventory (Romeo, Yepes, Berger, Guàrdia, & Castro, 2011). In the Identification‐Commitment Inventory need commitment involves retaining the job, as a medium of continuance and survival and involves questions on self‐reported turnover intention but answers are then reversed to capture commitment from a need perspective. Here the answers are not reversed to instead measure turnover intention. For example: “I don't like how this organization functions, I will go to a better one as soon as I can.” The 5‐point Likert‐response scale ranged from 1 (Strongly disagree) to 5 (Strongly agree). Internal consistency (*ω*) of the scale at T1 and T2 is reported in Table 1.

**Table 1:** Descriptive statistics, scale reliability and intercorrelations for all study variables

Variables	M	*SD*	1	2	3	4	5	6	7
1. Turnover Intention T1	2.47	0.91	(0.68)						
2. Overcommitment T1	2.47	0.80	0.19**	(0.84)					
3. Quality of Care T1	4.01	0.60	−0.43**	−0.35**	(0.85)				
4. Turnover Intention T2	2.62	0.92	0.61**	0.15*	−0.42**	(0.74)			
5. Overcommitment T2	2.43	0.82	0.09	0.61**	−0.25**	0.26**	(0.85)		
6. Quality of Care T2	4.03	0.61	−0.27**	−0.22**	0.58**	−0.45**	−0.33**	(0.84)	
7. Organizational Change T2	3.24	1.42	0.18**	0.09	−0.23**	0.25**	0.23**	−0.25**	(–)

Note

*N* = 226. *M* = Mean, *SD* = Standard Deviation, *p < .05, **p < .01. Scale reliability calculated using McDonald's *ω*.

#### 2.4.2 Overcommitment

Overcommitment was measured using the six‐item work overcommitment scale (Siegrist et al., 2004). An example item is: “As soon as I wake up in the morning, I think about work.” Responses were given on a 5‐point Likert‐scale ranging from 1 (very seldom or never) to 5 (very often). Internal consistency (*ω*) of the scale at T1 and T2 is reported in Table 1.

#### 2.4.3 Quality of care

Quality of care was measured using the five‐item quality of care scale (Westerberg & Tafvelin, 2014). The questions concern satisfaction with how clients/patients are treated, kept informed and their wishes respected. Questions also relate to how well the needs of the clients/patients are met and the overall satisfaction of given care. For example: “At my workplace I experience that enough consideration is taken of the clients’/patients’ opinions and wishes.” Responses were given on a 5‐point Likert‐scale ranging from 1 (very seldom or never) to 5 (very often or always). Internal consistency (*ω*) of the scale at T1 and T2 is reported in Table 1.

#### 2.4.4 Organizational change

Organizational change was measured using a single item: “Have you been affected by organizational changes during the last year?” Employees were thus reporting on changes that had taken place and had an impact on them prior to the current investigation. We adopted this single item from the organizational change literature, where it has been used as an independent variable in organizational change studies (Tvedt, Saksvik, & Nytr⊘, 2009). Responses were given on a 5‐point Likert‐scale ranging from 1 (not at all) to 5 (to a large extent).

### 2.5 Analysis

The statistical analyses were carried out using JASP 0.10.2 (JASP team, 2019) for descriptives and Mplus version 8.3 (Muthén & Muthén, 2019) for longitudinal *SEM*. Robust maximum likelihood estimation was used for all *SEM* analysis. Missing data were handled by using full information maximum likelihood estimation (Enders, 2010). Because the data were collected from different departments, approaches accounting for clustering of data could be warranted (Hox, Moerbeek, & Van de Schoot, 2017). To examine the variance attributed to between‐department variation, we retrieved intraclass correlations for all outcome variables and for the independent organizational change variable. Using the intraclass correlation values together with the average cluster size (i.e. employees per department) we then calculated design effects. As no design effects were above the suggested cut‐off level of 2 (Muthen & Satorra, 1995) and the number of departments relatively few (*N* = 18), the data were analysed using a single‐level approach (Hox et al., 2017).

Before testing the structural model, a longitudinal measurement model was specified using latent variables at T1 and T2 to ensure that the same underlying construct was measured over time (Little, 2013). Using a sequential approach with no constraints (configural), equality constraints on factor loadings (Metric), equality constraints on items intercept (scalar), Equality constraints on factor variances (Strict – fv) and finally also on residual error variances (Strict – rev). In line with Chen’s (2007) recommendations for evaluation of structural invariance across models, change in CFI (ΔCFI) of less than 0.01 and a change in RMSEA (ΔRMSEA) of less than 0.015 or a change in SRMR (ΔSRMR) of less than 0.03 (0.01 for scalar and strict) would support invariance across time. The residuals of the same items were allowed to covary over time in the specified longitudinal models since indicator‐specific variance is likely to correlate over time (Little, 2013).

Based on the measurement model a structural model was constructed. In the structural model turnover intention, overcommitment and quality of care at T2 were regressed on organizational change and on turnover intention, overcommitment and quality of care at T1 (to control for baseline levels of these factors). To evaluate the model fit recommended goodness‐ of‐ fit standards were used (Kline, 2015).

## 3 RESULTS

Descriptive statistics and correlations between all study variables are presented in Table 1. All variables at T1 and T2 were associated in expected directions, turnover intention and overcommitment was negatively associated with quality of care and turnover intention was positively associated with overcommitment. As expected, strong associations were also found between the same measures at T1 and T2 and organizational change was statistically significantly associated with all three outcomes in expected directions.

The test of longitudinal invariance supported a strict‐residual variance model, on which we then based the structural model. In the structural model Hypothesis 1–3 were tested (i.e. whether organizational change during the last year is associated with turnover intentions, overcommitment and perceived quality of care). Model fit results for the tested measurement models and the structural model (including organizational change) is presented in Table 2.

**Table 2:** Model fit of the longitudinal measurement models and the structural model

Model	*χ* ^2^	*df*	*p*	CFI	TLI	RMSEA [90% Cl]	SRMR
Configural	451.12	321	.00	0.95	0.94	0.04 [0.03 0.05]	0.06
Metric	469.02	332	.00	0.94	0.94	0.04 [0.03 0.05]	0.06
Scalar	479.20	343	.00	0.94	0.94	0.04 [0.03 0.05]	0.06
Strict‐fv	480.36	346	.00	0.94	0.94	0.04 [0.03 0.05]	0.06
Strict‐rev	495.01	361	.00	0.94	0.94	0.04 [0.03 0.05]	0.07
Structural	586.36	391	.00	0.92	0.92	0.05 [0.04 0.06]	0.10

**Figure 1:**
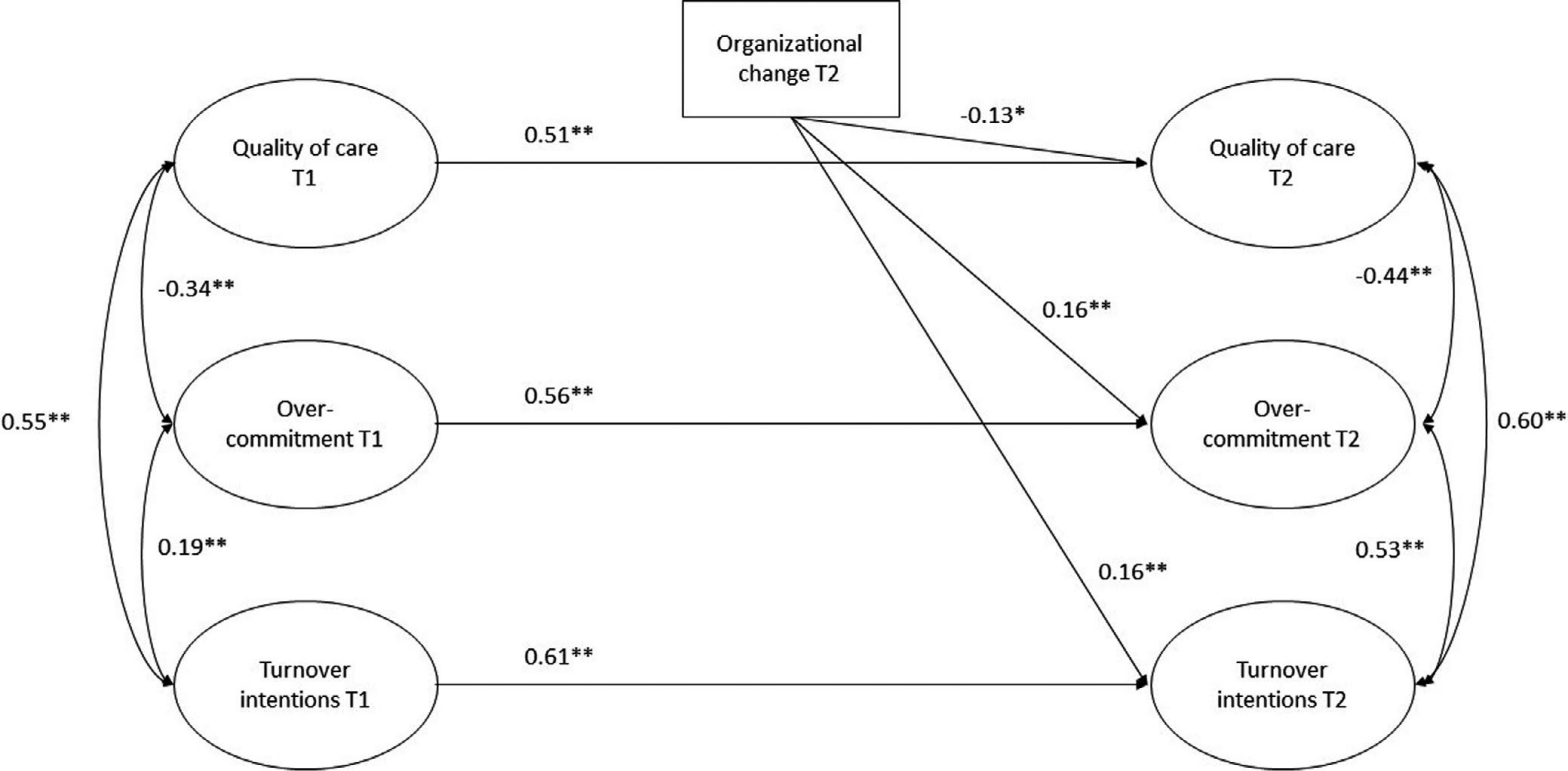
The longitudinal structural equational model

Overall, the structural model showed an acceptable fit to the data: *χ* ^2^ (391) = 586.36, *p* < .00, CFI = 0.92, TLI = 0.92, RMSEA = 0.05 [0.04 0.05], SRMR = 0.10. In line with Hypothesis 1, organizational change during the last year was positively associated with turnover intentions at T2 (*β *= 0.16, *p* < .01), controlled for turnover intentions at T1. With control for overcommitment at T1, organizational change during the last year was also positively associated with overcommitment at T2, (*β* = 0.16, *p* < .01), supporting Hypothesis 2. Support was also found for Hypothesis 3, as organizational change during the last year was negatively associated with quality of care at T2 (*β* = −0.13, *p* < .01), controlled for quality of care at T1. The structural model with paths and standardized coefficients is presented in Figure 1.

## 4 DISCUSSION

In the present study, we examined the association between organizational change and turnover intention, overcommitment and quality of care, among nurses and nursing assistants in an eldercare setting. In line with our hypothesis, the results of the study show statistically significant relationships between organizational change and all three outcomes in expected directions. Organizational change was positively associated with turnover intentions and overcommitment and negatively associated with employees’ perceived quality of care.

A sound work environment has a profound impact on eldercare employees’ willingness to remain employed and therefore factors contributing to a healthy workplace are important to consider (Clausen et al., 2014; Stone et al., 2017; Tourangeau, et al., 2014). The results of our study show that organizational change can interfere with objectives to create these healthy workplaces in eldercare organizations. Given the high frequency of organizational change initiatives in eldercare organizations (von Treuer et al., 2018), our results suggest that the consideration of how, when and why these changes are conducted may play an important role for of keeping the work environment sound.

Thus, as time to recover from physical and mental strain is often too short in eldercare under normal conditions (van der Borg et al., 2017), the addition of frequent changes may be seen as a tipping point for that forces employees to engage in coping to handle increased demands. In turn, this may lead to significant problems for already vulnerable eldercare organizations, as employees may choose to leave, becomes absent from work due to overcommitment leading to burnout and/or not being able to provide a high quality of care.

Putting the change processes on a better track through actions that facilitates more constructive coping mechanism and thereby reduce change‐induced threats to employee well‐being, can be one way of creating a healthier organizational change process. Saksvik et al. (2007), concluded that it is not the type of change, but rather how the change process is managed that influences how employees are affected by organizational change. Eldercare organizations may thus have higher levels of success in implementing change initiatives without unwanted negative employee outcomes when sufficient resources are in place. For example, when their work environment is perceived as innovative and when managers adopt a supportive leadership style (von Treuer et al., 2018).

Change initiatives using participatory approaches to managing change, where employees are involved as active agents has also been concluded as important for increasing success rates and reducing negative effects of organizational change (Abildgaard, Nielsen, & Sverke, 2018; Lundmark, von Thiele Schwarz, Hasson, Stenling, & Tafvelin, 2018). In eldercare settings, participatory job crafting activities, where employees drive change to elements of their job, has been concluded positively related to their well‐being (Romeo et al., 2019). Therefore, as there are no signs of the pace of organizational changes being reduced, paying more attention to how the they are managed seems warranted.

## 5 LIMITATIONS AND FUTURE RESEARCH

The present study entails several limitations which need considering before drawing firm conclusions from the results. First, although a longitudinal design is used, this applies to the controlling of outcomes in relationship to baseline. The studied relationships between organizational change and outcomes were cross‐sectional. Although not compensating for the lack of a fully longitudinal design, the question on organizational change differed from the other questions as the respondents were asked to respond retrospectively to changes during the last year. Also, the results of this study are in line with studies using longitudinal designs exploring similar relationships (de Jong et al., 2016). It has moreover been suggested that common method bias may be less of a threat to validity than once thought (Spector, 2006). Having said this, future studies should when possible strive for the use of a longitudinal design where dependent and independent variables are separated in time to enable stronger conclusions about the direction of the relationships.

*Second*, the response rate for the panel sample (i.e. those answering the questionnaire at both T1 and T2), was relatively low (36%) and a statistically significant difference was found between the panel sample and the baseline sample in terms of turnover intentions. However, response rates of approximately 40% are often found in naturalistic questionnaire‐based studies (Guo, Kopec, Cibere, Li, & Goldsmith, 2016) and the lower score on turnover intentions in the panel sample is reasonably explained by actual turnover between T1 and T2. Given that a lot can be gained in terms of validity by studying organizational change as it occurs, we believe that the present study in the light of similar findings in organizational change studies, adds information about potential consequences of organizational change in the specific eldercare context. In future, replicating studies that examine the relationship between organizational change and employee outcomes in healthcare settings should preferably aim at larger sample sizes and less drop‐offs between data collections, to draw stronger conclusions.

*Third,* a single‐item question asking participants to rate to what degree they had been affected by organizational change during the last year was used. Using a single item, instead of a scale to evaluate a phenomenon can be psychometrically questionable. Conversely, a recent study on the use of single‐item questions in organizational studies has shown that they can be considered as valid measures and be an effective way to shorten questionnaires for increased response rates (Fisher, Matthews, & Gibbons, 2016). Additionally, the question does not capture the respondents’ appraisals of the changes as positive or negative), nor their perceptions of prerequisites for implementing change (Nielsen, 2018). However, the fact that “affected by” may include both positive and negative appraisals indicates that even stronger relationships may be found between perceived unbeneficial change and undesirable outcomes. It may also indicate that no matter what kind of change (positive or negative) without sufficient resources, change may have deterioration effects. In future studies, including questions on employees’ positive or negative appraisals and perceptions of prerequisites for the change may give additional clues to how these perceptions relate to change coping and change outcomes.

## 6 PRACTICAL IMPLICATIONS

The results of our study suggest that the way organizational changes are implemented in eldercare is important to consider for maintaining a sound work environment and thus in the long run for the possibility to recruit and retain nurses and nursing assistants in these organizations. The study adds to the nursing literature interested in identifying factors that influence the work environment in care organizations. It also adds to the organizational literature interested in the relation between organizational changes and employee outcomes, showing that organizational change may be especially important to consider in eldercare organizations. Eldercare employees have a central role in the providence of high‐quality care for older people, a care sector projected to vastly increase over the coming years (Stone et al., 2017). Awareness of factors that contribute eldercare employees’ well‐being, willingness to remain in service, as well providing high‐quality care could therefore be considered a vital task for nurse managers. As reoccurring organizational changes seem to be an inevitable part of most eldercare organizations, we suggest that nurse managers allocate adequate resources to work with these organizational changes. We also suggest that the use of a supportive leadership style and a participatory approach to implement changes may improve outcomes as this will increase chances of a healthy change process (Abildgaard et al., 2018; von Treuer et al., 2018). Additionally, the current COVID‐19 pandemic has seriously affected employees and patients in Spanish and Swedish older people in care institutions in Spain and Sweden (Comas‐Herrera et al., 2020). Our results could help understand how recent extensive organizational changes may have played a part in this. Going into the pandemic with hampered employee resources to deal with the additional strain of preventing the spread of the disease may thus be one explanation for why the spread of the disease has been severe in this setting. It may thereby also contribute to the development future measures (e.g. making sure that employees’ have sufficient resources to manage additional strain) to counteract the potential effects of a future crises. More research on this subject is warranted.

## 7 CONCLUSION

We found a positive relationship between organizational change and, from an organizational perspective, the detrimental coping strategies turnover intention and overcommitment. We also found a negative relationship between organizational change and appraisal of quality of care, as an indicator of employees not managing to exert effort into both changes and keeping up with performing core work tasks. If not managed properly, organizational change meant to improve operations can result in negative staff consequences such as increased turnover and overcommitment and reduced possibilities to provide high quality of care. All which are known to negatively affect patient safety. Therefore, nurse managers need to consider how organizational change in eldercare can be managed for employees to cope with the increased strain that organizational changes may impose.

## CONFLICT OF INTEREST

None of the authors would like to report a conflict of interest.

## AUTHORS’ CONTRIBUTION

All authors contributed to the study conception and design. Data collection was performed by Montserrat Yepes‐Baldó and Marina Romeo (Spain), as well as Kristina Westerberg and Maria Nordin (Sweden). Robert Lundmark performed the data analysis and wrote all parts of the paper. All authors read, commented on and approved the final manuscript.

## ETHICAL APPROVAL

Research Ethics Committee approval was received from the University of Barcelona, Spain, and from the Regional Board of Ethics, Umeå, Sweden [2015‐62‐31Ö]. All participants were provided with information about the study prior to taking part. They also received information stating that their data would be analysed anonymously. All participants signed consent forms and had the option to opt out at any time.

## Data Availability

All results of data analysed during this study are included in this published article and its supplementary files. The data set analysed during the current study are available from the corresponding author on reasonable request.
